# Analysis of the technical-scientific production of the National Council for Scientific and Technological Development (CNPq) productivity fellows in Pediatrics

**DOI:** 10.31744/einstein_journal/2020AO5043

**Published:** 2019-12-10

**Authors:** Thaís Carolina Klepa, Bruno Pedroso

**Affiliations:** 1 Universidade Estadual de Ponta Grossa, Ponta Grossa, PR, Brazil

**Keywords:** Pediatrics, Scientific and technical activities, Scientific publication indicators, Researcher performance evaluation systems, Bibliometrics

## Abstract

**Objective:**

To analyze the technical-scientific production of research productivity fellows of the National Council for Scientific and Technological Development, in Pediatrics, from 2013 to 2016.

**Methods:**

First, data were obtained identifying fellowship researchers using the Lattes Platform, and subsequently calculating the indicators present in their Lattes curricula using scriptLattes software v8.10.

**Results:**

In the period studied, 17 fellowship researchers were identified. They published a total of 524 articles in journals, most of them ranked as high and intermediate Qualis. In addition, fellowship researchers conducted 158 supervisions during the period, published 119 books or chapters and 465 papers in conference proceedings.

**Conclusion:**

The Brazilian scientific production in Pediatrics has shown to be significant and of good impact, both nationally and internationally. However, the distribution of research groups is concentrated in specific regions of Brazil.

## INTRODUCTION

The triad comprised of science, technology and innovation has defining factors for social and economic development of regions and countries.^(^^1^^)^ This triad requires monitoring and financing from governments and funding agencies. Among the types of funding provided by the National Council of Scientific and Technological Development (CNPq - *Conselho Nacional de Desenvolvimento Científico e Tecnológico)* are fellowships for the development of researchers and their research.

In this scenario, productivity fellowships are initiatives with encouraging potential. These fellowships are considered rewards, acknowledging individuals with an exceptional role in developing scientific knowledge, and their recipients are part of a reference group within the Brazilian scientific academia.

The requirements to achieve the position of productivity fellow researcher of the CNPq are extremely demanding. There are two modalities of productivity fellowship: research, and technological development and innovation extension. Both have three categories: senior, 1, and 2.

Category 2 productivity fellowship is the initial modality, and the appointment into this class is based on appraisal of a candidate's productivity, focusing on published articles and supervision of theses and dissertations during the past five years. For category 1, the assessment takes into account the past 10 years. Category 1 has levels A, B, C and D, level A being the highest. Category senior is reserved for researchers that have excelled their peers as an exemplary leader and paradigm in their research fields. Category senior requires at least 20 years as Category 1 researcher, with 15 years at levels A or B.^(^^2^^)^

In addition to the general requirements by CNPq, such as showing a well-defined line of research, proposing research projects with scientific relevance and holding a ranking compatible with the available number of fellowships in the category, there are CNPq specific criteria to be attained by the different categories and levels regarding production and supervision for each area of knowledge.^(^^2^^)^

Although not an explicit requirement, engagement in *stricto sensu* graduate programs by productivity research fellows has been observed to be an essential factor.

Scientific production assessment of *stricto sensu* graduation programs in Brazil, is performed using the WebQualis system, created in 1998 and upgraded in 2008. WebQualis is a database generated by several procedures that were set up by the Higher Education Personnel Improvement Coordination (CAPES - *Coordenação de Aperfeiçoamento de Pessoal de Nível Superior*) and has provisions for each area of knowledge. WebQualis is based on the information made available by graduation programs through the *Sucupira Coleta/Plataforma* [Sucupira Collecting/Platform] application, aiming to rank scientific journals and measure the quality of the intellectual production of graduation programs. Its last upgrading has defined a ranking of eight strata: A1, A2, B1, B2, B3, B4, B5 and C.^(^^3^^)^

Although CAPES recommends not using WebQualis for other applications, it has been used for identifying knowledge produced in Brazil, becoming an assessment indicator for scientific research outcomes, definition of goals and resource distribution, such as is the procedure for granting productivity fellowships.

Considering the ideas briefly discussed up to now, it can be said that the assessment of scientific production in a given area of knowledge helps managers and researchers define the strategies for funding allocation, and to expand the understanding on areas that are lagging behind. There are fields of knowledge, such as bibliometrics and scientometrics, aiming to evaluate how science is produced. The former consists in the quantitative study of information, its production, promotion and use. Scientometrics studies the same aspects specifically related to science.^(^^4^^,^^5^^)^

Considering Pediatrics as a field of knowledge, few scientometric/bibliometric studies have been published aiming to evaluate its scientific production. One study assessed the period between 2010 and 2012 based on the CNPq Lattes platform, and concluded that 8.8% (47) of medical researchers performed research in Pediatrics, with a total of 1,174 published articles.^(^^6^^)^ Another study, from the period between 2011 and 2014, was not restricted to the medical area, showing 132 Pediatric research groups, with 14.4% from the non-medical knowledge area.^(^^7^^)^ In publications evaluating more extensive periods, when compared to international production, the Brazilian Pediatric scientific production showed an increase from 0.51% to 1.6% in total publications.^(^^8^^)^ Considering all periods assessed, higher production was observed from institutions located in the Southeastern region of Brazil. The different approaches in these studies make it difficult to establish a time trend progression of scientific production in Pediatrics.

## OBJECTIVE

Analyze the technical-scientific output of productivity fellows of the National Council for Scientific and Technological Development (CNPq), in the Pediatric medical area, from 2013 to 2016 in Brazil, based on the assessment of *stricto sensu* graduation that is performed by CNPq.

## METHODS

Initially, on September 14, 2017, we obtained a list of researchers from the Lattes Curriculum database, using the filters “Productivity fellows of CNPq” in all categories, and “Professional area” related to the broader Health Sciences area, Medicine, Internal Medicine sub-area, Pediatrics specialization. Then, after identification, curriculums of researchers were searched on the CNPq Lattes Platform. Considering the period studied (from 2013 to 2016), each curriculum was computed, by the scriptLattes software v8.10,^(^^9^^)^ on September 17, 2017.

The scientific production computed comprised the following indicators: full-text articles published in journals, papers published in conference proceedings, books and chapters, technical productions, arts productions and student supervision. Then, data were organized using Microsoft Excel^®^ software 2010, according to year of publication, fellowship category and, in the case of articles published in journals, the WebQualis 2013-2016 ranking, considering the highest ranking among all areas, and the ranking in the Medicine II area, which includes Pediatrics.

We decided to use both metrics for two reasons. First, for CNPq and CAPES, Pediatrics does not constitute a separate assessment area. Actually, CAPES assesses Pediatrics within the Medicine II area. Second, due to the limitations set by the previous item, the researchers included in the present study were identified by their area of activity described on their Lattes curriculum, and participation in graduation programs in Medicine II was not mandatory, as their engagement might be established with another area of assessment. The priority was a researcher's compliance with the metrics of graduation programs they were engaged in. We registered the highest ranking as universal metrics, valid for all areas.

Since the study was considered as an observational, analytic or retrospective description, it was exempted by the Ethics Research Committee (CEP) of *Universidade Estadual de Ponta Grossa*.

## RESULTS

**Figure 1:**
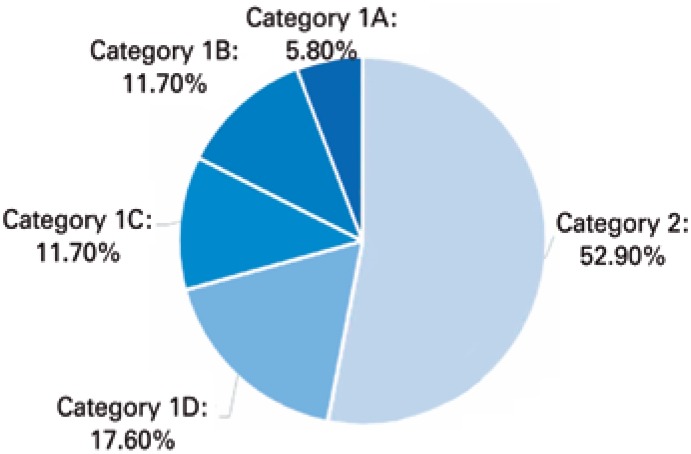
Distribution of Pediatric Productivity Fellowship categories of the National Council for Scientific and Technological Development

**Figure 2:**
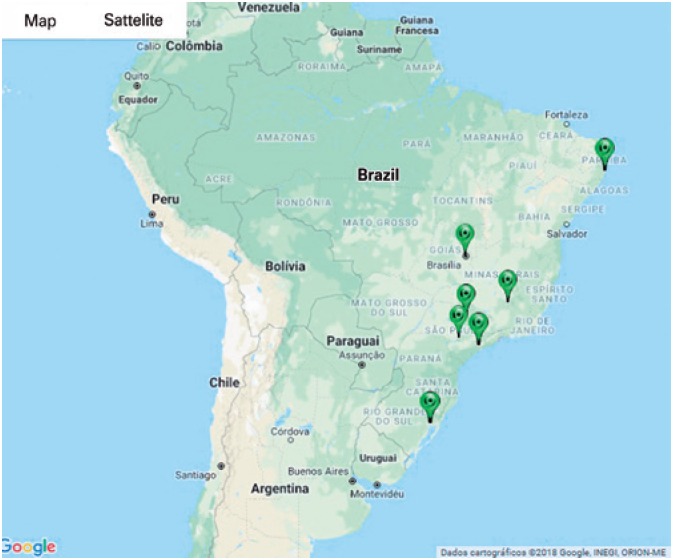
Map showing location of productivity fellows of the National Council for Scientific and Technological Development in Pediatrics

**Figure 3:**
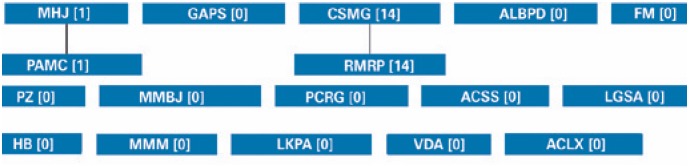
Inter-collaboration among productivity fellows of the National Council for Scientific and Technological Development in Pediatrics

The search using the Lattes platform identified 17 CNPq productivity fellows in Pediatrics. Mostly, they were classified as category 2 (52.9%) (Figure 1). The majority of researchers was female (76.4%), and the geographic distribution of Pediatric researchers was similar to the distribution for other areas of knowledge, showing a predominance of research centers from the Southern Region (Figure 2). Additionally, there was a low rate of collaboration among researchers (Figure 3).

During the period defined in the study (2013-2016), a total of 524 articles were published in journals, as shown in table 1. Considering the higher Qualis ranking, the largest proportion of articles came from the high strata (A1 e A2), without noticeable differences between the number of articles published in each year of the period assessed. On the other hand, when considering the Qualis ranking of the journals in the Medicine II area, there is a shift to the intermediate Qualis strata (B1 e B2).

**Table 1:** Distribution of total articles published according to the highest Qualis journal ranking and Qualis Medicine II journal ranking

	Highest				Medicine II			
	2013	2014	2015	2016	2013	2014	2015	2016
A1	47	43	54	38	6	12	10	13
A2	41	43	41	63	31	24	25	20
B1	12	24	14	21	33	39	38	43
B2	0	3	2	5	14	13	15	27
B3	6	4	0	5	10	22	17	13
B4	12	6	9	1	4	1	5	9
B5	0	0	0	1	12	5	6	3
C	2	0	2	0	2	0	2	0
N/I*	5	3	8	9	13	10	12	15
Total	125	126	130	143	125	126	130	143

* N/I: journals not indexed by Qualis.

When considering the distribution of publications among researchers, the predominance of upper strata in the WebQualis ranking remained, regardless of the productivity fellowship category. In addition, some fellows classified in lower fellowship categories (1D and 2) showed number and quality of articles published similar to those presented by higher category researchers (1A) (Table 2).

**Table 2:** Relation between researchers, productivity fellowship categories and number of articles published in journals according to the Qualis ranking

Researcher	Published papers according to the Qualis journal ranking									
	Rank	A1	A2	B1	B2	B3	B4	B5	C	N/I
1	1A	17	9	3	2	0	2	0	0	1
2	1B	33	26	2	0	1	1	0	1	2
3	1B	15	21	4	0	0	0	0	0	1
4	1C	2	12	2	0	0	16	0	0	0
5	1C	10	14	7	2	4	3	0	0	2
6	1D	22	7	2	0	0	0	0	0	1
7	1D	6	1	3	2	0	2	0	0	1
8	1D	10	9	6	0	0	1	0	0	2
9	2	15	34	16	0	0	1	0	0	7
10	2	6	20	7	1	3	0	0	0	1
11	2	10	11	4	0	2	0	0	0	0
12	2	6	0	2	0	2	0	0	0	0
13	2	6	5	5	1	1	0	0	1	1
14	2	9	4	4	0	0	1	0	0	4
15	2	2	7	0	0	1	0	1	1	0
16	2	7	5	0	1	1	0	0	1	1
17	2	8	7	5	1	0	1	0	0	1

N/I: journals not indexed by Qualis.

Among the main publication targets, we observe a high number of Brazilian and international journals (277), and those with the highest number of articles published by the research fellows are shown in table 3. With the exception of the journals with the highest number of articles published, the remaining journals presented satisfactory Impact Factors and they were indexed in extensively accessible databases.

**Table 3:** Major journals of Pediatric productivity fellows and Impact Factors

Journals	Articles (n)	Highest Qualis	Qualis Medicine II	JCR	Cite Score - Scopus	SJR – Scopus	SciELO
Brazilian Journal of Allergy and Immunology	19	B4	B5	–	–	–	–
*Arquivos Brasileiros de Cardiologia*	17	A2	B2	1.318	0.88	0.381	0.4296
*Jornal de Pediatria*	17	A1	B1	1.690	1.63	0.704	0.4152
Osteoporosis International	12	A1	A2	3.856	3.5	1.523	–
PLOS One	11	A1	A2	2.766	3.01	1.164	–
Journal of Rheumatology	10	A2	A2	3.470	2.63	2.157	–
*Jornal Brasileiro de Pneumologia*	8	A2	B2	1.532	0.96	0.448	0.4790
*Revista Brasileira de Reumatologia*	8	A2	B3	1.350	0.82	0.340	0.2397
*Revista Paulista de Pediatria*	8	B1	B3	–	0.9	0.472	0.4632
The Pediatric Infectious Disease Journal	8	A2	B1	2.305	2.01	1.392	–

JCR: *Journal Citation Reports*; SJR: Scimago Journal Rank.

During the period analyzed researchers contributed to human resources training by means of 158 supervisions completed, comprising 46 Undergraduate Research Mentorship Programs, one Course Completion Paper, 54 Master's degrees, 53 PhD degrees and four Post-PhDs, with little variation in the number of supervisions in the years studied (Table 4).

**Table 4:** Supervision completed per researcher

Researcher	Supervision completed					
	Post-PhD	PhD	MSc	Specialization	CCP	URMP
1	2	4	0	0	0	1
2	1	7	2	3	0	5
3	0	4	6	0	0	4
4	1	2	2	0	0	0
5	0	1	4	0	0	2
6	0	1	1	0	0	1
7	0	3	3	2	0	0
8	0	1	1	0	0	0
9	2	13	4	0	0	7
10	0	2	1	1	0	3
11	0	2	0	0	0	0
12	0	1	1	0	1	0
13	0	3	10	0	0	7
14	0	4	3	0	0	0
15	0	4	5	0	0	7
16	0	3	4	0	0	28
17	0	2	7	0	0	0

CCP: Course Completion Paper; URMP: Undergraduate Research Mentorship Programs.

We observed 461 abstracts published in conference proceedings, but only four were published as full texts. The literature production showed 108 book chapters, seven books and four collections. Technical production consisted of only 65 in the period studied, with 33 technical papers – largely, manuals.

## DISCUSSION

We can see the relevance and impact of the scientific production in Pediatrics in Brazil. In comparison to the study of Gonçalves *et al.,*^(^^6^^)^ there was a reduction in the number of active fellows. In contrast, the geographical distribution of scientific research headquarters in Pediatrics remained stable, underscoring the need for geographic expansion in research centers in the area. However, unlike the author's results, there was a predominance of female fellows (76.4%).

In this area of knowledge, a low rate of inter-collaborations among productivity fellows was observed. Assessment of co-authorships among productivity fellows allows for the inference that there is the development of multicenter studies on certain topics, in addition to enabling exchange of information among the different research centers. Thus, it is relevant to foster the establishment of collaboration networks among researchers in the area.

Albeit the low number of fellow researchers, they produced several scientific quality papers. The CNPq productivity fellowship categories were not highly related to the number and impact of publications, and lower category researchers had a performance similar to upper categories, in regard to publication of articles.

Journals with the highest numbers of articles published by these fellows, with rare exceptions, had a high Impact Factor, both at the national and international level, which highlights Brazilian research in Pediatrics at the global level.^(^^8^^)^

The academic activities of leaders also focused on the training of new researchers, with a predominance in Master's and PhD supervisions, and less supervisions for undergraduates.

In contrast to the performance in scientific article publication, in general, fellows have a low index of scientific literature work, such as books and collections. However, the number of book chapters was noteworthy for the period studied, and when added to technical work, help meet the objective of reporting knowledge.

It is pertinent to underscore that using WebQualis for purposes diverse from the one it was developed for has generated criticism to the system, mainly due to the increasing exclusion of Brazilian journals, to failure to predict the quality of scientific publications and to non-existing standardization of assessment metrics.^(^^10^^,^^11^^)^

Among the frailties attributed to the WebQualis system, the methodology shows: lack of presentation of inductive character to compare to publications of distinct areas, restricted use of journals that have attained publication of articles in the previous year, use of the Impact Factor as the only metric and, last, absence of criteria for assessing publications outside the specific area.^(^^10^^)^

The way in which assessment methods are being used by funding agencies to measure productivity of graduate programs and of researchers, makes the process degrading,^(^^3^^)^ ao passo since it places faculty in a chain of writing and publishing, instead of generating relevant knowledge to a specific area of knowledge.

Even if the study objective did not encompass the analysis of the merit of publications, researchers have been observed to becoming more and more frequently mere “writers”. In the current graduation assessment system, faculty has been replaced by researchers, who publish articles in journals afterwards indexed, and read exclusively by researchers themselves.^(^^3^^)^ Even if unconsciously, researchers and institutions have involved themselves in obstinate scientific production, aimed to generate points for assessment of graduate programs. In this scenario, scientific journals adapt to the assessment system of funding agencies in effect, assuming the new requirements and needs of universities, expanding the number of peer reviewers, increasing the number of issues, and pursuing indexations, Impact Factor and high rankings on CAPES’ WebQualis.

The fact that productivity fellows comprise a lean group in relation to the total number of researchers in Pediatrics in Brazil is a limitation of the study, and thus the present study was not able to reflect the scenario of the area. However, the objective was not to analyze the area as a whole, given that the comprehensive mapping of such researchers would be inaccurate, no matter the methods adopted. Thus, we only studied a portion, which theoretically represents the “upper edge” of this universe.

## CONCLUSION

Pediatrics, as a field of knowledge, faces difficulties in scientific production, possibly due to the scarcity of medical professionals who dedicate themselves to scientific research, and to challenging ethical factors related to the development of clinical trials for this age group. However, Brazilian scientific production in Pediatrics has been positive, with a high number of domestic and international publications in high impact journals.

The reduction in the number of research productivity fellows in Pediatrics, at CNPq, observed in the present study, provides evidence of the need for ongoing fostering of scientific production. In addition, the poor regional distribution of researchers remained the same, underscoring the need for creating research groups in the remaining regions of the country, and encouragement for inter-collaboration among different researchers.

## References

[1] 1. Rocha EM, Ferreira MA. Indicadores de ciência, tecnologia e inovação: mensuração dos sistemas de CTeI nos estados brasileiros. Cienc Inf. 2004; 33(3):61-8.

[2] 2. Conselho Nacional de Desenvolvimento Científico e Tecnológico (CNPq). RN-028/2015 [Internet]. Brasília (DF): CNPq; 2015 [citado 2019 Jun 3]. Disponível em: http://www.cnpq.br/web/guest/view/-/journal_content/56_INSTANCE_0oED/10157/2958271

[3] 3. Barata RC. Dez coisas que você deveria saber sobre o Qualis. RBPG. 2016; 13(30):13-40.

[4] 4. Macias-Chapula CA. O papel da informetria e da cienciometria e sua perspectiva nacional e internacional. Cienc Inf. 1998;27(2):134-40.

[5] 5. Silva JA, Bianchi ML. Cientometria: a métrica da ciência. Paidéia. 2001; 11(21):5-10.

[6] 6. Gonçalves E, Santos MI, Maia BT, Brandão RC, Oliveira EA, Martelli Júnior H. Produção Científica dos Pesquisadores da Área de Pediatria no CNPq. Rev Bras Educ Med. 2014;38(3):349-55.

[7] 7. Oliveira PH, Pinheiro MG, Isquierdo LA, Sukiennik R, Pellanda LC. Brazilian pediatric research groups, lines of research, and main areas of activity. J Pediatr (Rio J). 2015;91(3):299-305. 10.1016/j.jped.2014.09.002 25431857

[8] 8. Blank D, Rosa LO, Gurgel RQ, Goldani MZ. Brazilian knowledge production in the field of child and adolescent health. J Pediatr (Rio J). 2006;82(2):97-102. Review. 10.2223/JPED.1454 16614762

[9] 9. Mena-Chalco JP, Cesar Junior RM. ScriptLattes: an open-source knowledge extraction system from the Lattes platform. J Braz Comput Soc. 2009; 15(4):31-9.

[10] 10. Rocha-e-Silva M. O novo Qualis, ou a tragédia anunciada. Clinics. 2009; 64(1):1-4.10.1590/s1807-5932200900010000119142543

[11] 11. Rocha-e-Silva M. Qualis 2011-2013 – os três erres. Clinics. 2010;65(10):935-6. 10.1590/S1807-59322010001000001 PMC3008861 21120289

