# Supplementary material for: Analysis of the technical-scientific production of the National Council for Scientific and Technological Development (CNPq) productivity fellows in Pediatrics
Source: Einstein (Sao Paulo). 2019 Dec 10;18:eAO5043. doi: 10.31744/einstein_journal/2020AO5043 (PMC6910066; doi:10.31744/einstein_journal/2020AO5043)
Supplement: Translation

# Translation: Análise da produção técnico-científica de bolsistas de produtividade do Conselho Nacional de Desenvolvimento Científico e Tecnológico (CNPq) na área da Pediatria

**Authors:** Thaís Carolina Klepa, Bruno Pedroso

**Affiliations:** 1 Universidade Estadual de Ponta Grossa, Ponta Grossa, PR, Brasil

**Keywords:** Pediatria, Atividades científicas e tecnológicas, Indicadores de produção científica, Sistemas de créditos e avaliação de pesquisadores, Bibliometria

## Abstract

**Objetivo:**

Analisar a produção técnico-científica de bolsistas de produtividade em pesquisa do Conselho Nacional de Desenvolvimento Científico e Tecnológico, na área de Pediatria, no período de 2013 a 2016.

**Métodos:**

Os dados foram obtidos identificando-se os bolsistas, por meio da Plataforma Lattes, e, posteriormente, contabilizando-se os indicadores presentes em seus currículos Lattes, pelo software scriptLattes v8.10.

**Resultados:**

No período, foram identificados 17 pesquisadores bolsistas, os quais publicaram 524 artigos em periódicos, em sua maioria classificados com Qualis elevado/intermediário. Ainda, os pesquisadores realizaram, no período, 158 orientações, publicaram 119 livros/capítulos e 465 trabalhos em anais de congressos.

**Conclusão:**

A produção científica brasileira na área de Pediatria mostrou-se expressiva e de impacto, em âmbito nacional e internacional. Entretanto, a distribuição dos grupos é concentrada em regiões específicas do Brasil.

## INTRODUÇÃO

A tríade formada por ciência, tecnologia e inovação constitui fatores diferenciadores do desenvolvimento social e econômico de países e regiões.^(^^1^^)^ Isso requer acompanhamento e incentivo por parte dos governos e agências de fomento. Dentre os incentivos de fomento ofertados pelo Conselho Nacional de Desenvolvimento Científico e Tecnológico (CNPq), estão as bolsas oferecidas para o desenvolvimento do pesquisador e de suas pesquisas.

Nesse contexto, as bolsas de produtividade são iniciativas cujo potencial é considerado promissor. A estas tem sido atribuído o *status* de prêmio, em reconhecimento por uma atuação de destaque na construção do conhecimento, sendo os detentores dessas bolsas constituintes de um grupo de referência no meio acadêmico brasileiro.

As exigências para se alcançar o posto de pesquisador bolsista de produtividade do CNPq são bastante elevadas, existindo duas modalidades de bolsa de produtividade: em pesquisa e em desenvolvimento tecnológico e extensão inovadora. Em ambas as modalidades, há as categorias sênior, 1 e 2.

A categoria 2 é a inicial. A seleção é pautada pela avaliação da produtividade, enfatizando os trabalhos publicados e as orientações nos últimos 5 anos. Na categoria 1, a avaliação é pautada pelo últimos 10 anos, e há as subdivisões entre os níveis A, B, C e D, sendo o nível A o mais elevado. A categoria sênior é reservada para pesquisadores que se destaquem entre seus pares como líder e paradigma em sua área de atuação, sendo requerido permanecer bolsista por, pelo menos, 20 anos na categoria 1, sendo 15 destes no nível A ou B.^(^^2^^)^

Além das exigências gerais do CNPq, como possuir linha de pesquisa definida, apresentar projeto de pesquisa de mérito científico, e atingir classificação compatível com a cota de bolsas disponíveis na categoria, há ainda critérios específicos para as diferentes categorias e níveis quanto à produção e às orientações para cada área do conhecimento.^(^^2^^)^

Ainda que não constitua exigência explícita, tem se observado que a vinculação dos bolsistas de produtividade a programas de pós-graduação *stricto sensu* é fator imperativo.

A avaliação da produção científica dos programas de pós-graduação *stricto sensu* no Brasil, por sua vez, ocorre por meio do sistema WebQualis, criado em 1998 e ajustado em 2008. Ele se constitui de uma base de dados elaborada por meio de diversos procedimentos, estabelecidos pela Coordenação de Aperfeiçoamento de Pessoal de Nível Superior (CAPES), sendo específico para cada área do conhecimento. Baseia-se nas informações disponibilizadas pelos programas de pós-graduação, por meio do aplicativo Coleta/Plataforma Sucupira, tendo como objetivos a classificação dos periódicos científicos, além de pretender mensurar a qualidade da produção intelectual dos programas de pós-graduação. Em sua última atualização, ficou definida uma classificação com oito estratos, sendo eles: A1, A2, B1, B2, B3, B4, B5 e C.^(^^3^^)^

Não obstante a recomendação da CAPES para que o WebQualis não seja utilizado para outros fins, este tem sido, com frequência, utilizado para a identificação do conhecimento produzido no Brasil, constituindo indicador para avaliação dos resultados da pesquisa científica, estabelecimento de metas e direcionamento de recursos, tal qual ocorre no processo de concessão das bolsas de produtividade.

Frente a esse breve introito, a avaliação da produção científica em determinada área do conhecimento auxilia gestores e pesquisadores, na definição de estratégias para a distribuição dos recursos financeiros e ampliação do conhecimento em áreas defasadas. Com o objetivo de avaliar a forma como a ciência vem sendo produzida, existem ramos do conhecimento que abordam tal perspectiva, dentre as quais podem-se citar a bibliometria e a cientometria. A primeira consiste no estudo quantitativo da informação, sua produção e disseminação/uso, enquanto a cientometria estuda esses aspectos relacionados à ciência em específico.^(^^4^^,^^5^^)^

Considerando a Pediatria como área do conhecimento, poucos estudos cientométricos/bibliométricos foram publicados, no intuito de avaliar sua produção científica. Um dos estudos acerca do tema avaliou o período entre 2010 e 2012 e se baseou na plataforma Lattes do CNPq, concluindo que 8,8% (47) dos pesquisadores da área médica realizaram pesquisa na área pediátrica, levando a um total de 1.174 artigos publicados.^(^^6^^)^ Outro estudo, referente ao período entre 2011 e 2014, não se restringiu à área médica, resultando em 132 grupos de pesquisa em Pediatria, dos quais 14,4% enquadraram-se em outra área do conhecimento, que não a médica.^(^^7^^)^ Em publicações com período de avaliação mais abrangente, a avaliação comparativa com a produção mundial permitiu concluir um crescimento da produção científica brasileira em Pediatria de 0,51% a 1,6% no total de publicações.^(^^8^^)^ Em todos os períodos de avaliação, observou-se maior número de produções advindas de instituições sedisadas na Região Sudeste do Brasil. As diferenças das abordagens nesses estudos dificultam o estabelecimento do padrão de evolução temporal da produção científica em Pediatria.

## OBJETIVO

Analisar a produção técnico-científica dos bolsistas de produtividade do Conselho Nacional de Desenvolvimento Científico e Tecnológico (CNPq), cuja temática fosse Pediatria e a área de atuação a Medicina, no quadriênio 2013-2016, seguindo o período de avaliação da pós-graduação *stricto sensu* no Brasil, realizado pelo CNPq.

## MÉTODOS

Inicialmente, a lista dos pesquisadores foi obtida por busca de Currículo Lattes realizada com os filtros “Bolsistas de produtividade do CNPq” em todas as categorias e “Atuação Profissional” referente à grande área de Ciências da Saúde, área de Medicina, subárea de Clínica Médica e especialidade em Pediatria, realizada em 14 de setembro de 2017. Assim, os pesquisadores foram identificados e tiveram seus currículos buscados na Plataforma Lattes do CNPq. Considerando o período de interesse da pesquisa (2013 a 2016), os currículos foram contabilizados pelo software scriptLattes v8.10,^(^^9^^)^ em 17 de setembro de 2017.

A produção científica contabilizada consistiu dos seguintes indicadores: artigos completos publicados em periódicos, trabalhos publicados em anais de congressos, livros e capítulos, produções técnicas, produções artísticas e orientações. Posteriormente, os dados foram tabulados usando-se o software Microsoft Excel^®^ 2010, de acordo com o ano de publicação, a categoria da bolsa e, no caso dos artigos publicados em periódicos, a classificação no WebQualis 2013-2016, sendo considerada a maior classificação entre todas as áreas e a classificação na área de Medicina II, a qual abarca a Pediatria.

A opção pela utilização de ambas as métricas justificou-se pelos fatos de (1) a Pediatria não constituir área de avaliação do CNPq ou da CAPES − sendo, nesta última, abarcada pela área da Medicina II, e (2) tendo em vista que, devido à limitação citada no item anterior, os pesquisadores objetos de estudo da presente investigação foram identificados por sua área de atuação descrita no currículo Lattes, sem constituir obrigatoriedade a participação em programas de pós-graduação da área da Medicina II, podendo o vínculo ocorrer com outras áreas de avaliação e sendo a prioridade destes cumprir as métricas da área específica dos programas de pós-graduação em que estão inseridos, considerou-se a maior classificação como métrica universal, válida para todas as áreas.

Por se tratar de estudo observacional, analítico ou descritivo retrospectivo, o artigo foi isento pelo Comitê de Ética em Pesquisa (CEP) da Universidade Estadual de Ponta Grossa.

## RESULTADOS

A busca na Plataforma Lattes identificou a existência de 17 bolsistas de produtividade do CNPq atuantes na área de Pediatria. Em sua maioria, foram classificados na categoria 2 (52,9%) (Figura 1). Houve predominância do sexo feminino (76,4%) nessa área, e a distribuição geográfica dos pesquisadores em Pediatria foi semelhante a demonstrada em outras áreas do conhecimento, com predomínio de centros de pesquisa na Região Sudeste (Figura 2). Ainda, esses bolsistas de produtividade apresentaram baixo índice de intercolaborações (Figura 3).

**Figura 1:** Distribuição entre as categorias de Bolsas de Produtividade do Conselho Nacional de Desenvolvimento Científico e Tecnológico em Pediatria

**Figura 2:** Mapa de geolocalização dos bolsistas de Produtividade do Conselho Nacional de Desenvolvimento Científico e Tecnológico na área de Pediatria

**Figura 3:** Gráfico de intercolaborações dos bolsistas de produtividade do Conselho Nacional de Desenvolvimento Científico e Tecnológico em Pediatria

No período considerado pela pesquisa (2013-2016), foram publicados 524 artigos em periódicos, conforme pode ser visualizado na tabela 1. Considerando a maior classificação no Qualis, a concentração esteve em seus estratos superiores (A1 e A2), sem diferença visível entre o número de artigos publicado em cada ano no período estudado. Em contraposição, quando considerada a classificação Qualis dos periódicos na área da Medicina II, houve deslocamento para estratos intermediários do Qualis (B1 e B2).

**Tabela 1:** Distribuição do total de artigos publicados em relação ao maior Qualis e ao Qualis da área de Medicina II dos periódicos

	Maior				Medicina II			
	2013	2014	2015	2016	2013	2014	2015	2016
A1	47	43	54	38	6	12	10	13
A2	41	43	41	63	31	24	25	20
B1	12	24	14	21	33	39	38	43
B2	0	3	2	5	14	13	15	27
B3	6	4	0	5	10	22	17	13
B4	12	6	9	1	4	1	5	9
B5	0	0	0	1	12	5	6	3
C	2	0	2	0	2	0	2	0
N/I*	5	3	8	9	13	10	12	15
Total	125	126	130	143	125	126	130	143

* N/I: periódicos não indexados na Qualis.

Ao considerar a distribuição das publicações entre os pesquisadores, o predomínio de estratos superiores da classificação da WebQualis se manteve, independente da categoria de bolsa de produtividade. Além disso, alguns bolsistas classificados em categorias inferiores de bolsa (1D e 2) apresentaram número e qualidade de artigos publicados semelhantes aos apresentados por pesquisadores de maior categoria (1A) (Tabela 2).

**Tabela 2:** Relação entre pesquisadores, nível de bolsa de produtividade e número de artigos publicados em periódicos segundo a classificação Qualis

Pesquisador	Artigos publicados de acordo com Qualis do periódico									
	Nível	A1	A2	B1	B2	B3	B4	B5	C	N/I
1	1A	17	9	3	2	0	2	0	0	1
2	1B	33	26	2	0	1	1	0	1	2
3	1B	15	21	4	0	0	0	0	0	1
4	1C	2	12	2	0	0	16	0	0	0
5	1C	10	14	7	2	4	3	0	0	2
6	1D	22	7	2	0	0	0	0	0	1
7	1D	6	1	3	2	0	2	0	0	1
8	1D	10	9	6	0	0	1	0	0	2
9	2	15	34	16	0	0	1	0	0	7
10	2	6	20	7	1	3	0	0	0	1
11	2	10	11	4	0	2	0	0	0	0
12	2	6	0	2	0	2	0	0	0	0
13	2	6	5	5	1	1	0	0	1	1
14	2	9	4	4	0	0	1	0	0	4
15	2	2	7	0	0	1	0	1	1	0
16	2	7	5	0	1	1	0	0	1	1
17	2	8	7	5	1	0	1	0	0	1

N/I: periódicos não indexados na Qualis.

Entre os principais alvos de publicação, esteve um extenso número de periódicos (277), tanto nacionais quanto internacionais, sendo os com maior número de artigos publicados pelos bolsistas citados na tabela 3. Com exceção dos periódicos com maior número de artigos publicados, os demais apresentaram Fatores de Impacto satisfatórios e estavam indexados em bases de dados de amplo acesso.

**Tabela 3:** Principais periódicos dos bolsistas de produtividade em Pediatria e Fatores de Impacto

Periódicos	Artigos (n)	Maior Qualis	Qualis Medicina II	JCR	Cite Score - Scopus	SJR - Scopus	SciELO
*Brazilian Journal of Allergy and Immunology*	19	B4	B5	–	–	–	–
Arquivos Brasileiros de Cardiologia	17	A2	B2	1.318	0,88	0,381	0.4296
Jornal de Pediatria	17	A1	B1	1.690	1,63	0,704	0.4152
*Osteoporosis International*	12	A1	A2	3.856	3,5	1,523	–
PLOS One	11	A1	A2	2.766	3,01	1,164	–
*Journal of Rheumatology*	10	A2	A2	3.470	2,63	2,157	–
Jornal Brasileiro de Pneumologia	8	A2	B2	1.532	0,96	0,448	0.4790
Revista Brasileira de Reumatologia	8	A2	B3	1.350	0,82	0,340	0.2397
Revista Paulista de Pediatria	8	B1	B3	–	0,9	0,472	0.4632
*The Pediatric Infectious Disease Journal*	8	A2	B1	2.305	2,01	1,392	–

JCR: *Journal Citation Reports*; SJR: *Scimago Journal Rank.*

Os pesquisadores contribuíram com a formação de recursos humanos, por meio de 158 orientações concluídas no quadriênio, sendo 46 de Iniciações Científicas, 1 Trabalho de Conclusão de Curso, 54 mestrados, 53 doutorados e 4 pós-doutorados, com pouca variação no número de orientações entre os anos investigados (Tabela 4).

**Tabela 4:** Orientações concluídas por pesquisador

Pesquisador	Orientações concluídas					
	Pós-Doutorado	Doutorado	Mestrado	Especialização	TCC	IC
1	2	4	0	0	0	1
2	1	7	2	3	0	5
3	0	4	6	0	0	4
4	1	2	2	0	0	0
5	0	1	4	0	0	2
6	0	1	1	0	0	1
7	0	3	3	2	0	0
8	0	1	1	0	0	0
9	2	13	4	0	0	7
10	0	2	1	1	0	3
11	0	2	0	0	0	0
12	0	1	1	0	1	0
13	0	3	10	0	0	7
14	0	4	3	0	0	0
15	0	4	5	0	0	7
16	0	3	4	0	0	28
17	0	2	7	0	0	0

TCC: Trabalho de Conclusão de Curso; IC: Iniciação Científica.

Foram publicados 461 resumos em anais de congressos, mas apenas 4 foram publicações do texto completo. A produção literária contou com 108 capítulos de livros, 7 livros e 4 coletâneas. As produções técnicas se limitaram a apenas 65 no período considerado, sendo 33 trabalhos técnicos − em sua maioria, manuais.

## DISCUSSÃO

É possível observar a relevância e o impacto da produção científica em Pediatria no Brasil. Em comparação com o estudo de Gonçalves et al.,^(^^6^^)^ houve redução no número de bolsistas ativos. Já a distribuição geográfica das sedes de pesquisa científica em Pediatria se manteve, evidenciando a necessidade da ampliação geográfica dos centros de pesquisa nessa área. Entretanto, ao contrário do apresentado pelo autor, houve predominância feminina entre os bolsistas (76,4%).

Nesta área do conhecimento, foi observado baixo índice de intercolaborações entre os bolsistas de produtividade. A avaliação de coautorias entre os bolsistas de produtividade permite concluir a elaboração de estudos multicêntricos em determinados temas, além de possibilitar a troca de informações entre os diferentes centros de pesquisa. Assim, é relevante incentivar a configuração de redes de colaboração entre os pesquisadores da área.

As publicações de artigos científicos entre os pesquisadores foram numerosas e de qualidade, mesmo considerando o baixo número de bolsistas. As categorias de bolsas de produtividade do CNPq apresentaram pouca relação com a quantidade e o impacto das publicações, e pesquisadores de categorias inferiores apresentaram desempenho semelhante aos de categorias superiores, no que tange às publicações de artigos.

Os periódicos que apresentaram os maiores números de artigos publicados por esses bolsistas, com raras exceções, apresentam alto Fator de Impacto, tanto a nível nacional quanto internacional, o que coloca a pesquisa em Pediatria brasileira em evidência em nível mundial.^(^^8^^)^

A formação de novos pesquisadores também foi colocada em foco nas atividades acadêmicas desses líderes, com predomínio para orientações de mestrado e doutorado, e com menor número de orientações ao nível da graduação.

Contrapondo-se ao desempenho na publicação de artigos científicos, de maneira geral, os bolsistas apresentaram baixo índice de produções literárias, como livros e coletâneas. Todavia, o número de capítulos de livros foi considerável para o período estudado e, somando-se às produções técnicas, ajuda a cumprir o objetivo de disseminação do conhecimento.

É pertinente ressaltar que a utilização do WebQualis para finalidades distintas daquela para qual foi desenvolvido tem gerado críticas ao sistema, principalmente no que se refere à exclusão cada vez mais acentuada das revistas brasileiras, na insuficiência para predizer a qualidade das publicações científicas e pela inexistência de padronização nas métricas de avaliação.^(^^10^^,^^11^^)^

Entre as fragilidades creditadas ao sistema WebQualis, na metodologia, há: falta de apresentação do caráter indutivo na comparação de publicações de áreas distintas, utilização restrita de periódicos que obtiveram publicações de artigos no ano anterior, uso do Fator de Impacto como métrica única e, por fim, ausência de critérios para avaliar publicações fora da área específica.^(^^10^^)^

Da forma como os métodos de avaliação estão sendo empregados pelas agências de fomento para medir a produtividade dos programas de pós-graduação e pesquisadores, o processo torna-se degradante^(^^3^^)^ ao passo que insere o professor em um ciclo para redigir e publicar, ao invés de gerar conhecimentos relevantes para determinada área do conhecimento.

Ainda que o objetivo da pesquisa não tenha compreendido a análise de mérito das publicações, tem se observado que, cada vez com maior frequência, os pesquisadores tornam-se meramente “escritores”. Diante do sistema de avaliação da pós-graduação vigente, o docente foi substituído pelo pesquisador, que publica artigos em periódicos indexados posteriormente e lidos exclusivamente pelos próprios pesquisadores.^(^^3^^)^ Mesmo que inconscientemente, pesquisadores e instituições se envolvem com a produção científica desmedida, com o objetivo de gerar pontuação para a avaliação da pós-graduação. Neste contexto, os periódicos científicos se adaptam a esse meio de avaliação vigente das agências de fomento, admitindo novas demandas e necessidades das universidades, ampliando o número de revisores, aumentando o número de edições, e batalhando por indexações, Fator de Impacto e uma boa classificação no WebQualis da CAPES.

Constitui limitação do estudo o fato de os bolsistas de produtividade formarem um grupo enxuto em relação ao total de pesquisadores da área da Pediatria no Brasil, sabendo-se que a presente investigação não foi capaz de refletir o cenário da área. Entretanto, não se objetivou analisar a área como um todo, tendo em vista que o mapeamento da íntegra de tais pesquisadores, quaisquer que fossem os métodos adotados, seriam imprecisos. Dessa forma, trabalhou-se somente com um recorte que representa, em tese, o “bordo superior” deste universo.

## CONCLUSÃO

A Pediatria, enquanto área do conhecimento, enfrenta dificuldades na produção científica, possivelmente devido à escassez de profissionais da área médica que se dedicam à pesquisa científica, e aos fatores éticos dificultadores relacionados à elaboração de ensaios clínicos nessa faixa etária. Entretanto, a produção científica brasileira em Pediatria tem se mostrado positiva, com grande número de publicações no âmbito nacional e internacional, em periódicos de alto impacto.

A redução do número de bolsistas de produtividade em pesquisa no CNPq na área de Pediatria, no presente estudo, evidencia a necessidade do incentivo constante ao crescimento da produção científica. Ainda, a distribuição regional pífia dos pesquisadores se manteve, reforçando a necessidade da criação de grupos de pesquisa nas demais regiões do país, além do incentivo à intercolaboração entre os diferentes pesquisadores.
